# Update on Surgical Management of Pediatric Urolithiasis

**DOI:** 10.3389/fped.2019.00252

**Published:** 2019-07-03

**Authors:** Sajid Sultan, Sadaf Aba Umer, Bashir Ahmed, Syed Ali Anwar Naqvi, Syed Adibul Hasan Rizvi

**Affiliations:** Philip G. Ransley Department of Paediatric Urology, Sindh Institute of Urology and Transplantation, Karachi, Pakistan

**Keywords:** urolithiasis, minimally invasive surgery, extracorporeal shockwave lithotripsy, endoscopic intracorporeal lithotripsy, retrograde intrarenal surgery, percutaneous nephrolithotomy, robotics, pediatric urolithiasis

## Abstract

Urolithiasis has always been a fascinating disease, even more so in children. There are many intriguing facets to this pathology. This article is a nonsystematic review to provide an update on the surgical management of pediatric urolithiasis. It highlights the pros and cons of various minimally invasive surgical options such as extracorporeal shockwave lithotripsy (ESWL), retrograde intrarenal surgery (RIRS), percutaneous nephrolithotomy (PCNL), laparoscopy, and robotics. This article also describes the various intracorporeal disintegration technologies available to fragment the stone, including the newer advancements in laser technology. It also emphasizes the cost considerations especially with reference to the emerging economies. Thus, this manuscript guides how to select the least-invasive option for an individual patient, considering age and gender; stone size, location, and composition; and facilities and expertise available.

## Introduction

Urolithiasis is still intriguing due to its mysterious and complex nature, although being known to mankind from before the Christian era. Urolithiasis presents in all age groups including children from the neonatal period onward and may even be picked up on prenatal ultrasound (1). Apart from a high prevalence of pediatric urolithiasis in endemic areas (2, 3), there is an increasing incidence all over the world (4). Within the pediatric age group, clinical presentations are varied, and evaluation, including imaging, and management have to be modified depending on whether they are infants, preschool children, or pre- or post-pubertal teenagers (2, 5–7).

Pediatric patients carry a high probability of recurrence (8), and therefore, every effort should be made to prevent stone recurrence by ensuring complete stone clearance (9). This is a greater challenge with minimally invasive surgery (MIS) and limits the applicability of extracorporeal lithotripsy. Risk factors need to be identified, and these may be anatomical or metabolic, which require evaluation by dietary, urinary, and stone composition analyses (6, 10–14).

Historically, all stones are treated by open surgery (15–17). Currently, with the advent of MIS, the majority of the stones are managed by MIS utilizing extracorporeal shockwave lithotripsy (ESWL), percutaneous nephrolithotomy (PCNL), and ureteroscopy/retrograde intrarenal surgery (URS/RIRS) (2, 18, 19). Undertaking MIS in small children is challenging (20), which to a certain extent has been overcome by improvement in technology by the development of miniaturized instruments, which are referred to appropriately as Miniperc or Microperc (21–28). Sophisticated fine-caliber rigid and flexible ureteroscopes with improved optics are now available (29), which together with the development of high-power laser disintegration technology (30–32) and fine retrieval instruments such as nitinol baskets and graspers (33) have revolutionized MIS in children.

There are many facets to urolithiasis. In this review article, we will focus on the current status of the surgical management options available and attempt to guide the reader how to choose the best option considering the age, gender, stone size, location, and composition; the available expertise and facilities; considerations of cost; and both the surgeon's and patient's preferences.

## Methods

For this nonsystematic review, a comprehensive search of the PubMed database was performed. This literature review is a survey focused on recent articles on surgical management of pediatric urolithiasis, which included the following key words; ESWL, PCNL, MiniPerc, MicroPerc, URS, RIRS, laparoscopy, and robotics in pediatric urolithiasis. This will also include our experience of surgical management of pediatric urolithiasis for the last two decades.

## Extracorporeal Shockwave Lithotripsy

Since 1986, when ESWL was first described, it remains the first-line of treatment of renal stones in children (34). However, its scope is now being challenged for the reasons that there have not been any major improvements in ESWL technology to improve the ESWL outcome of renal stone management, and it has largely failed to keep pace and compete with the better outcomes now being reported from the newer MIS developments such as RIRS and mini/micro PCNL.

### Technical Aspects of ESWL

The fundamental prerequisite for a successful ESWL is that the shockwaves can pass into the body and hit the stone with minimal loss of energy. In contrast to the pioneer lithotripter HM3 (with water bath), the later lithotripters have a gap between the shockwave source (therapy head) and the body that has to be bridged with a liquid transmission medium (usually an ultrasound gel) without any trapped air bubbles.

Devices using video cameras have been developed to check if the transmission zone is free of air bubbles and coupling is optimal (35). This equipment has been recently incorporated in the therapy head of some lithotripters with obvious advantage.

Another improvement has been the improved identification of the target and maintenance of an optimal position of the patient throughout the procedure by the use of continuous fluoroscopy or real-time ultrasound with lower radiation exposure (36).

ESWL in children are already being performed under general anesthesia, and therefore, high-frequency shallow ventilation may be used to reduce the range of respiratory movement and consequently increase the hit rate.

The risks of tissue injuries and subsequent bleeding can be reduced by inducing vasoconstriction by starting ESWL treatment with a series of shockwaves at a low energy level, followed by a pause or continuing with the low-power shockwaves for a longer period but incorporating a stepwise increase in the power. This treatment modality is termed as *ramping*. Ramping has many other advantages such as identifying the minimum energy level at which a stone starts to disintegrate, maintaining an optimum energy level which overcomes the attractive forces between the stone crystals, and avoiding overtreatment in terms of the energy level deployed. Several studies have shown that ramping results in better disintegration than when power is rapidly increased to high levels or when a high power level is used constantly during the treatment. High energy levels result in larger stone fragments with higher chances of ureteric obstruction, and low energy levels result in smaller fragments which are easy to pass (37, 38).

ESWL may be performed at various frequencies ranging from 30 to 120 shockwaves/min. On the basis of clinical and experimental studies, the recommended shockwave frequency for children is 60/min (1 Hz) (35).

### Complications of ESWL

#### Obstruction

The most frequent problem following ESWL is ureteric obstruction caused by impacted stone fragments. Its frequency increases with a large stone burden and when the stone is disintegrated into larger fragments. Steinstrasse is the commonest presentation where stone fragments accumulate in the distal ureter and cause severe obstruction. The reported incidence of abdominal colic is 6.29% and steinstrasse 6.0 and 8.5% (18, 39). This can be avoided by the placement of an internal stent preoperatively.

#### Infection

Urinary tract infection is another common complication which can occur secondary to infected urine or stone. This can be avoided by the use of preoperative antibiotics according to the culture and sensitivity reports and being insistent that preoperative urine cultures are confirmed as sterile.

#### Subcapsular Hematoma and Collateral Injury

Particular attention must be paid to the, fortunately, rare risk of renal subcapsular hematoma, renal parenchymal injuries, and injuries to the surrounding structures. One case of significant perirenal hematoma and one case of small asymptomatic subcapsular hematoma and skin bruising have been reported (40, 41). One of the major causes of subcapsular hematoma in children is the use of an excessive number of shockwaves and/or unnecessarily high energy levels in order to try to fragment the stone in a single session. It is always better to stop and re-treat. Successive ESWL sessions should have an interval of at least 10–14 days. Calcium channel blockers, various antioxidants, and free-radical scavengers have been used to decrease these injuries especially in retreatment cases (35).

To carry out lithotripsy in a safe and harmonious way, it is appropriate to avoid excessive numbers of shockwaves and high-risk energy levels. Considering these factors, repeated ESWL is not considered a failure but a simple consequence of the physics behind noninvasive stone disintegration (42–44). That may be true for adults where ESWL is mostly performed without general anesthesia and as an outpatient procedure. However, in children, there is the additional factor that most ESWL are performed under general anesthesia (or sedoanalgesia in bigger children) in order to avoid apprehension, pain, and movement and to keep the stone under the shockwave target. In children, repeated ESWL carry a bigger burden which needs to be factored into the equation.

There is wide disparity in the stone free rates documented in the literature for ESWL in children ranging from 33 to 95% with incomplete information about the number of sessions (retreatment rates), residual fragments, and JJ stent placements. The results are incomparable not just for lack of information but also because of variability of the confounding factors like age, gender, the stone burden, location, composition, the type of lithotripter used, and the number of shockwaves in each session. All these factors impact ESWL outcome especially in children (39, 45–47).

### Stone Burden

Although staghorn stones (>3 cm) have been treated with ESWL, the retreatment rates are high, requiring up to five sessions (48). The European Association of Urology (EAU)/European Society for Paediatric Urology (ESPU) and American Urological Association (AUA) guidelines continue to recommend ESWL as the first line of treatment for renal stones <2 cm, but the retreatment rates could still be high (49) and nomograms reflecting this have been developed (50, 51). Our recommendation is to restrict the use of ESWL for renal stones up to 1.5 cm, except for lower calyceal stones when the upper limit should be 1 cm.

In our study of pediatric ESWL, the results showed an increase in the mean number of ESWL sessions with an increase in stone burden. In the single stone group of 158 renal units with a mean stone size of 10 ± 2.5 mm, 76% (121 renal units) were cleared in a single session, and 21 units were cleared in a second session, giving an overall clearance rate of 89% in a mean of 1.14 sessions. In the second group of 58 renal units comprising multiple stones having a mean size of 17 ± 5.3 mm, 46% (27 units) were cleared in a single session, 32% (19 units) were cleared in a second session, and 7% (4 units) cleared in a third session, giving an overall clearance rate of 86% in a mean of 1.54 sessions (5).

### Stone Location

Lower calyceal stones have a poorer clearance rate as compared to renal pelvic, upper, and mid calyceal stones (52). Lower calyceal stone clearance by ESWL is dependent on anatomical factors such as lower calyceal infundibular width, length, and infundibulopelvic angle (53, 54).

In our pediatric ESWL data, we reviewed the relationship between stone location and clearance, which showed that the best clearance rates following a single session of ESWL were for the upper calyx (87%) and pelvis (84%), and the poorest for the lower calyx (67%). The mean numbers of sessions required for clearance in the upper calyx, pelvis, and middle calyx were 1.1, 1.2, and 1.3, respectively, as compared to the figure for the lower calyx of 1.5(5).

### Stone Composition

Cystine and calcium oxalate monohydrate (COM) stones are hard, dense stones and difficult to fragment by ESWL, therefore resulting in poor stone clearance. One of the methods for the identification of the stone composition is by the attenuation value [in Hounsfield units (HU)] (55) on a noncontrast computed tomography scan (NCCT), which can easily differentiate between calcium (*HU* > 1,000) and noncalcium stones (*HU* < 700). However, it will require an NCCT in every patient and therefore increased radiation hazards. A crude way of identifying a calcium stone (COM) on an X-ray kidney, ureter, and bladder (KUB) is a densely radiopaque stone, which matches with the density of bone in a vertebral body, while the pure noncalcium stones (uric acid; xanthine; 2,8-dihydroxyadenine) are nonopaque. These are fragile and therefore easy to fragment with ESWL. A cystine stone is faintly opaque with a ground glass appearance. Struvite stones generally present as staghorn stones and are slightly less opaque. Although struvite are soft stones which are easy to fragment with ESWL, any residual fragment has a high affinity for rapid stone regrowth and recurrence (56).

In a small study of 58 renal units, we assessed the relationship between the stone densities in Hounsfield unit and the results of treatment by ESWL. The outcome showed that a lower HU is associated with better clearance for the same size of stone (5). Similar observations had been documented by others (57).

## Endoscopic Intracorporeal Lithotripsy Disintegration Technologies

The current technology of intracorporeal lithotripsy provides the urologist with several effective options for stone disintegration depending on the type of the endoscope used [ureterorenoscope (URS) or nephroscope, rigid or flexible] and the location and accessibility of the stone.

Four stone disintegration technologies are available for intracorporeal lithotripsy during endoscopic management of urolithiasis (58, 59). Each device has certain unique properties that make it more suitable for a particular application. Manufacturers' claims may contain elements of bias, and therefore, a thorough and impartial evaluation is important in order to be able to select the most appropriate device in any particular situation.

### Electrohydraulic Lithotripsy

Although electrohydraulic lithotripsy (EHL) is the least costly and can be used even with flexible ‘scopes, it is relatively the most traumatic intracorporeal lithotripsy and is seldom used now.

### Ballistic Lithotripsy (Pneumatic Lithoclast)

Ballistic lithotripsy provides a durable, reusable, safe, and cost-effective means for stone fragmentation. It may be especially advantageous when large and hard stones are encountered. It can be used with scopes down to 10FG (Mini Perc) (21, 60, 61). Disadvantages could be a higher rate of stone repulsion, and it can only be used with rigid scopes.

### Ultrasonic Lithotripsy

It is safe, and although it may cause mucosal stripping, it does not create deeper perforations. For effective stone disintegration, it requires a relatively larger scope (>18FG) with a 4.5FG working channel to accommodate the hollow probe. It works best with standard PCNL for a large stone burden. It is less effective than pneumatic lithoclast (PL) for hard stones.

### Combination Devices

A PL combined with an ultrasonic beam aims to combine the advantages of both technologies. The superior, fast, coarse fragmentation ability of the pneumatic component is complemented by the simultaneous fine fragmentation and aspiration of the fragments via the hollow ultrasonic probe. Each modality can be activated separately or in unison. There are various types of the devices like Lithoclast Ultra (Boston Scientific, Natick, MAJ) and Cyber Wand (Olympus Surgical, Centre Valley, PA).

### Holmium: YAG Laser

This has brought great versatility to endoscopic intracorporeal lithotripsy (EIL) by introducing pulsed laser and allowing safe and effective stone fragmentation in the entire urinary tract. The laser output (power) can be adjusted by modulating the laser characteristics of energy (PE) and frequency (Fr). There are various generations of laser machines ranging from low power ( ≤ 20 W) to high power (120 W). The later machines allow for much more adjustment in PE and Fr, thus allowing the stone to be disintegrated into fragments (high PE, low Fr) or converted into dust/powder (low PE, high Fr). The fragments can be removed by baskets, and dust/powder exits with the irrigation fluid without the need for retrieval devices.

Some newer machines also have the ability to change the pulse length and pulse duration or pulse width. In this way, the same amount of power (PE × Fr) can be delivered in ultra-short pulse (150 ms) to extreme long pulse (800 ms). It is being highlighted that the ultra-short pulse has the advantage of more ablative power, but the long pulse produces less fiber degradation and less retropulsion and produces smaller residual fragments and promotes a more dusting technique (30–32).

The new high-power (120 W) machines have evolved with some innovative integrated and modulated technological modes such as the “Moses effect” and the “burst mode.” In the Moses effect, the laser pulse is divided into two phases; the first part divides the water between the laser fiber tip and stone, allowing the second part of the pulse to hit the stone unobstructed, thus being more ablative and less retropulsive (62). In the novel burst lasertripsy, each burst consists of three individual laser pulses having successively increasing pulse lengths, the first pulse being more energy intense while the last one is the least intense. It is suggested that the burst mode is significantly more ablative at similar power energy settings than the usual single pulse (63). Attempts are being made to incorporate a real-time stone/tissue differentiation capability using autofluorescence, thereby preventing the laser from firing against any structure other than the stone surface (64). Similar efforts are being made for the development of *in vivo* analysis of urinary stone composition (65). The manufacturers are trying hard to make a well-designed interface to make these holmium: YAG laser (Ho-YAG) laser lithotripters user-friendly.

Thulium laser technology has evolved and is gaining attention now that it is capable of pulsed emission. In comparison to Ho-YAG lithotripsy, it is two to four times faster and produces minimal or no retropulsion without any significant heat production. Therefore, pulsed thulium laser appears to have promising prospects (66).

## Retrograde Intrarenal Surgery

Since the development of sophisticated, miniaturized, and actively deflectable flexible ureterorenoscope (FLURS) with excellent optics and Ho-YAG lasers, RIRS has become a popular modality to treat upper ureteric and renal stones ≤2 cm (26, 67, 67, 68). FLURS with greater flexibility, maneuverability, secondary deflection capability, and wide range of deflection allows better treatment and access to lower pole urolithiasis (69, 70). However, in some cases, especially in younger children, it may be relatively difficult because of compromised deflection in renal pelvis. Stones > 2 cm and staghorn stones confer a high risk for treatment failure, illustrating that stone location and stone burden are the most important risk factors for treatment failure in RIRS as well.

A randomized trial comparing the outcomes of RIRS and mini PCNL in pediatric patients with stones >2 cm revealed that the success rate was significantly higher for mini PCNL with figures of 71 and 95%, respectively (60).

One of the advantages of RIRS is that urologists are used to performing procedures through the natural route of the urinary tract, which results in a short learning curve. RIRS is less invasive than PCNL and is therefore the most preferred approach to treat renal calculi in patients with a bleeding diathesis.

However, there are certain limitations with RIRS, especially in small children. The majority of the RIRS series, especially where the results are compared with PCNL, are biased by the fact that the children treated by RIRS are somewhat older and the stone burden is generally less than the PCNL groups (61, 71–74). A recent systematic review of children undergoing RIRS reported an aggregated success rate of 87.5% and a complication rate of 10.5%. However, the younger children had a much higher risk of complications (24%) compared with the older children (7%) (75). This clearly indicates that RIRS should be recommended with caution in younger children.

Children have narrow caliber ureters, and access is difficult or impossible without active dilatation with a balloon or preferably by inducing passive dilatation with prestenting. Concerns about ureteral ischemia, perforation, stricture formation, and vesicoureteric reflux as a result of dilating small caliber ureteric orifice are well recognized (76, 77). Although some studies do report access without prestenting in up to 60% of cases, active ureteral dilatation with 8–10 coaxial dilators were used in 97% of these cases. In the remaining 40% with failure to access, the pediatric ureter remains narrow and inaccessible for the URS at the ureteral orifice, the iliac vessels, or the uretereopelvic junction. Therefore, passive dilatation with prestenting was undertaken (77). It is also documented that prestenting does allow for more reliable access to the ureter and a shorter operative time.

Active or passive ureteric dilatation is also needed for the majority of the procedures, which required the placement of a 9/11FG ureteric access sheath (UAS). The UAS is placed to allow the FLURS to be removed and reintroduced repeatedly, allowing dust and fragments to clear and maintain good vision. The UAS also allows irrigation fluid to flow easily and so maintains a low intrarenal pressure, thereby decreasing the chances of pyelovenous and pyelolymphatic backflow and reducing the chances of developing sepsis.

UAS carries the risk of ureteric injury ranging from minimum mucosal damage to major lacerations, stricture, and avulsion (76). Therefore, almost all cases require post RIRS JJ stent placement for 2 weeks in order to allow the ureteric damage to heal. The majority of the series of RIRS in children which document the outcome from a single operative session do not take into consideration the anesthesia required for prestenting to provide passive ureteral dilatation. Similarly, since the majority of cases require postoperative stenting, especially where an UAS was used, they will require another anesthesia session for stent removal. Therefore, the outcome of RIRS in these patients is really that of a staged procedure requiring two to three general anesthetics and is not really a genuine single session.

### Ureterorenoscopes

URS is the treatment of choice for calculi, particularly in the distal and mid ureter and is more efficient than ESWL (78). Semi-rigid URS of size 4.5/6, 6/7.5, and 8/9.8FG are used depending on the age and anatomy of the patient and the size and location of the stone, as well as considering the technical requirements. The semirigid URS are more durable and have better visibility, faster irrigation flow, and larger working channels than the fully flexible models, and therefore, it is possible to access the whole ureter, even as far as the pelvicalyceal system. However, the ability of the ‘scope to bend is limited, and, especially with large psoas muscles, access to the upper ureter may be difficult in comparison with flexible URS. Therefore, semirigid URS may not be the first choice for the proximal ureteric stone (79).

The deflectable tip of the flexible ‘scopes is more suitable for a tortuous ureter and for upper ureteric stones, which often migrate into the kidney. The flexible ‘scope can follow the stones, and RIRS may be possible in the same session. The flexible ureteroscope permits only lasertripsy; they are costly and fragile, and therefore, the treatment with FLURS is much more expensive than with semirigid ‘scopes.

Several studies have highlighted the fact that the flexible ‘scope undergoes wear and tear and requires major repair after 14–32 RIRS sessions. Even without apportioning the high initial cost of the instrument, the maintenance and repair costs over each session of RIRS still make it a highly expensive treatment option (80, 81).

Lower ureteric stones are best managed by semirigid URSs by employing the PL or Ho-YAG laser (LL). The outcome in terms of stone clearance and complication rates is excellent and comparable. Although PL has the advantage of being significantly cheaper as compared to LL, PL can only be used through 6/7.5FG ‘scopes and above, while LL can be used with the small ‘scopes (4.5/6FG), which is an advantage for infants and small children (2, 5, 79, 82, 83).

Our results in 554 children with lower ureteric stones managed endoscopically using PL, low-power 30-watt Ho-YAG laser, or high-power 80-watt Ho-YAG laser performed between 2009 and 2016 with a comparable demography and stone burden showed excellent stone clearance rates of 91 vs. 89 vs. 95%, respectively (*p* = 0.5), with the number of sessions required showing no statistical difference between the three techniques. There was no difference in complications, which were Clavien I and II. However, it was recognized that the LL technique was costly.

Despite the minimally invasive nature of RIRS/URS, they are not without intraoperative and postoperative complications. A systematic review of 34 studies from 1996 to 2016 comprised of 2,758 children (2,994 procedures). A complication rate of 11.1% (327/2,994) was reported; 69% of these were Clavien grade I, and 31% were grade II/III. There were no Clavien grade IV and V complications (84). In another multicenter study of 642 children where semirigid ureterorenoscopy was performed for ureteric stones, a total of 54 (8.4%) complications have been documented where operative time was the only statistically significant parameter affecting the complication rate (78).

## Percutaneous Nephrolithotomy

### Patient Positioning

#### Prone Position

Percutaneous Nephrolithotomy was conventionally performed in the prone position for historical reasons, being more familiar to the majority of urologists and for fear of splanchnic injury. It also gives a wide operative field for manipulation of instruments and the possibility of access through multiple calyces (85, 86). Prone PCNL has certain limitations such as difficulty for the anesthetist with regard to control of the endotracheal tube and freedom of ventilation. Resuscitation is extremely difficult if an emergency such as cardiac arrest occurs during the procedure (87).

#### Supine Position

The first supine PCNL was performed in an adult by Valdivia-Uria in 1987 (88). Since then, many modifications of the supine position have been introduced to widen the operative field and increase maneuverability of the instruments, which were limitations of the original Valdivia-Uria supine position. Apart from that, most urologists were not familiar with the supine PCNL position. In most cases, the only access site available is the lower calyx, and there is hypermobility of the kidney, which needs to be controlled. There are number of potential advantages of supine pediatric PCNL. It is comfortable for the surgeon as he is working in a sitting position and it counters the anesthetic limitations inherent in the prone position.

In supine PCNL, the tract is horizontal or with a slight upward inclination in relation to the operative table, which allows most irrigation fluid and stone fragments to fall away from the patient, thus decreasing the risk of hypothermia and ensures a low pressure in the pelvicalyceal system, which results in a decreasing incidence of sepsis and better stone-free rates with fewer residual fragments. The operative time is usually shorter as time is saved by avoiding the need to change position following induction and quick clearance of the stone fragments (85, 89). A simultaneous endoscopic combined intrarenal surgery (ECIRS) can be performed particularly when the stone fragments are inaccessible with PCNL (90).

#### Lateral Position

Percutaneous Nephrolithotomy in a lateral position was first performed in a morbidly obese patient in 1994. Later on, more studies described the anesthetic advantages of lateral PCNL in the morbidly obese or kyphotic patients and patients with severe medical risk factors and comorbidities. They also describe the advantage of being able to rotate the C-arm fluoroscope to obtain an anteroposterior projection and perform nephroscopy simultaneously. Lateral PCNL has also been performed with ultrasound-guided renal access to reduce the radiation dose. The effectiveness of stone clearance in lateral PCNL lies in good ergonomics. The position of the pelvicalyceal system relative to the calyx enables gravity-assisted migration of calculous fragments from the calyces to the pelvis for easy removal. Conversely, there is a higher likelihood of stone fragment migration into the ureter, which may require preprocedural stenting or postprocedure ureterorenoscopy to remove the remaining ureteric stones. A number of other lateral PCNL techniques have been proposed, including the split leg modified lateral technique (85, 91). The operating time, stone clearance rates, and safety of lateral PCNL are comparable to those of prone and supine PCNL, but one of the disadvantages of lateral PCNL is that synchronous bilateral PCNL is impossible (85, 91).

### “Standard to Mini PCNL: How Small One Should Go?”

Classical (standard) PCNL in children required a 30FG Amplatz sheath and employed a 24FG nephroscope. The advantage of such generous access was very high (>90%) stone clearance rates in a single session but was not easily applicable in small children, where there was the risk of renal damage or excessive bleeding requiring blood transfusion.

The miniaturization of equipment for PCNL in pediatric patients has facilitated its use in all age groups and has also provided an opportunity to treat smaller stones that would otherwise be candidates for ESWL or RIRS. PCNL remains the gold standard treatment for large renal stone >2 cm and complex stones (49, 74).

Retrospective comparative studies have indicated that mini PCNL provides at least similar stone-free rate for moderate-size stones in comparison to RIRS (61). ElSheemy et al. also demonstrated superior results with mini PCNL (14FG) for renal calculi of 10–25 mm in preschool children in comparison to ESWL with comparable complication rates but a longer hospital stay (52).

In a large retrospective multi-institutional cohort including 1,205 pediatric renal units who underwent PCNL, the use of a sheath size >20FG was an independent predictor of complications and bleeding necessitating transfusion (92), and the association between a larger tract and greater blood loss has been confirmed in several other reports (25, 93, 94). Consistent with these reports, it was demonstrated that PCNL with a smaller tract <24FG results in lower blood loss without a decrease in success rate (18).

Similarly, in our study of 1,135 renal units, we have also observed that blood transfusion requirement was significantly less in all age groups when <20FG sheath was used. Blood transfusion rate was higher in children with larger stone burdens and in younger children with lower allowable blood loss (2).

The other important factors to prevent bleeding are an understanding of the pelvicalyceal and intrarenal vascular anatomy and the skilled application of a proper technique. The selection of the puncture site should be according to the stone location. The puncture is performed through the fornix, in the direction of the infundibulum to avoid trauma to the blood vessels adjacent to the infundibulum. In addition, while manipulating the nephroscope during stone fragmentation and retrieval, it is important to avoid excessive torque and the creation of false passages which traumatize the parenchyma.

There is still no strict standardized nomenclature for PCNL. Various classifications have been proposed and published in the literature, which include standard/conventional PCNL (22–30 FG), mini PCNL (11–22 FG), minimally invasive PCNL (MIP) (9.5–26 FG), Chinese mini PCNL (14–20 FG), ultra mini PCNL (11–13 FG), micro PCNL (4.8 FG), mini micro PCNL (8 FG), and super mini PCNL (10–14 FG). The first documented mini PCNL was by Jackman using an 11 FG peel away vascular access kit (95). Since then with the growing diversity of the miniaturized PCNL, the terminology mini PCNL has been loosely used for tract size 11–22 FG, and thus, mini PCNL is poorly defined (96–100).

We also initially started off with adult-sized nephroscopes (27 and 24FG) and gradually reduced it to 20, 18, and 15FG. Currently, we routinely use mini PCNL utilizing a 12FG nephroscope with a straight channel and offset lens or a 9.8FG cystoscope through a 16FG working sheath in most situations (2).

We occasionally employ rigid pediatric cystoscopes 6/7.5–10.5FG through a 12FG Amplatz sheath. These may be called an ultra mini PCNL. These scopes are readily available in most pediatric urology units and are sturdy, rigid, and reusable and, therefore, cost-effective. These ‘scopes have an added advantage for use in the narrow and difficult anatomy of small children and could be an economic compromise to microperc.

### Microperc

The term microperc refers to a system in which the telescope, working channel, and irrigation are combined in a needle, which can be as small as 4.8FG and requires only a single puncture, thereby avoiding the need for tract dilatation (101). The main advantage of microperc in children is to minimalize bleeding.

The technique may only be used in very selected cases, but it allows direct puncture into the relevant calyx via the all-seeing needle and direct fragmentation/powdering of the stone *in situ*. With no need for tract dilatation, less radiation exposure, and, consequently, less operating time, it should result in lower complication rates.

There are certain limitations of the microperc; first, the vision gets compromised more quickly with the slightest bleeding and stone dust. The vision may not be as good as in mini PCNL because the irrigation fluid is pushed intermittently, and there is no regular outflow passage. Necessary precautions should be taken to control the intrarenal pelvic pressure at every step of the procedure by placing a large caliber ureteric catheter in the renal pelvis, and saline irrigation should be carefully monitored during the procedure. It has been demonstrated that the intrarenal pressure is significantly higher in the micro PCNL compared to the standard PCNL (102).

Secondly, if the stone fragments migrate into a different calyx, it becomes impossible or difficult to access them. Thirdly, microperc instruments are costly and meant to be disposable (98), making their universal use in emerging economies, where much of the stone burden in children lies, extremely difficult.

The use of very small tracts has to be individualized on the basis of stone location and burden and balanced against the limitation of low irrigation fluid flow, impaired visualization, and limitation in the use of disintegration technology and grasping forceps.

The complications of PCNL in children have been reported from 9 to 27.7% (92, 97). Studies on micro PCNL (tract size <10 FG) reported a complication rate of 9% with a higher incidence of renal colic. Studies on ultra mini PCNL (tract size 10–14 FG) reported 14% complications with higher incidence of hematuria, renal extravasation, or renal pelvic perforation. A multi-institutional study of 1,157 children treated with PCNL (nephroscope 17 FG to 26 FG and URS 9.5 FG) reported a complication rate of 27.7%, where 7.7% were intraoperative and 20% were postoperative complications. The majority of the complications were Clavien grade I and II, and there were no grade IV and V complications (92, 97). Most of the series have limited numbers; therefore, risk factor analysis is difficult.

### Overview

One of the approaches to PCNL may be to start with a small caliber tract and, if needed, depending on the stone burden and its response to disintegration, then under direct vision to undertake progressive dilatation, keeping the risks of bleeding as low as possible. Alternatively, two or multiple smaller tracts may be established in order to achieve complete stone clearance if the stones are in places which are difficult/impossible to reach through the primary tract. In situations where the rigid instruments fail, the flexible nephroscope can be deployed in conjunction with lasertripsy.

The truism remains that one should try to keep the tract as small as possible but make it as big as needed.

## Endoscopic Combined Intrarenal Surgery

This is a combined procedure where both RIRS and PCNL are performed simultaneously by two surgeons working together. It may be performed either in the supine or prone position, but it is a situation where experience in supine PCNL clearly has benefits. ECIRS is usually recommended for complete clearance of a large stone bulk, such as a staghorn calculus and for multiple stones located in difficult anatomical positions. Such complex stones cannot be cleared either by RIRS or PCNL alone in a single session. It also reduces the operating time. The most appropriate renal puncture and the PCNL tract can be acquired easily with the assistance of ureterorenoscopy and especially if combined with ultrasound (103). There are still limited data on the outcomes of ECIRS in children (22, 104).

## Laparoscopic and Robotic Surgery

### Pyelolithotomy and Nephrolithotomy

With the advent of newer MIS modalities, the role of open surgery for urolithiasis has been minimized. With the increasing use of robotic surgery in pediatric urology, these approaches are being revisited. Laparoscopic and robotic-assisted pyelolithotomy (RPL) or nephrolithotomy (RPNL) or ureterolithotomy should not be the initial treatment choice for renal or ureteric stones. However, in selected patients, it can be a reasonable and safe minimally invasive surgical option even in children. Compared with a pure laparoscopic approach, robotic-assisted laparoscopic surgery has the advantages of improved dexterity for suturing and reconstruction (105, 106). It can be performed retroperitoneally; however, the transperitoneal approach is preferred by most surgeons. Compared to other endourological options, it has certain advantages of complete stone removal without fragmentation, thereby increasing the chances for complete stone clearance up to 96% in one procedure, especially when a single stone is present (107–110). It may prove particularly relevant for larger stones where the chances of residual fragments are high with other endoscopic procedures such as PCNL or RIRS. It has a significantly lower rate of bleeding and sepsis as has been shown in meta-analysis (111). Robotic pyelolithotomy and nephrolithotomy may be recommended in urolithiasis with concurrent pelvi-ureteric junction obstruction, where simultaneous reconstruction and repair are required. There are many unusual and difficult circumstances which may also prove amenable to the robotic-assisted approach (112):

° A very large stone burden, such as complete or partial staghorn calculi
° Stones containing gas
° A stone in a calyceal diverticulum
° Stones in ectopic kidneys
° Stones with complex urinary tracts with unfavorable collecting systems
° Failed previous endourological procedures
° Complex stones (especially very hard stones which are difficult to fragment) where multiple tracts for PCNL might otherwise be needed.

In practice, the majority of cases that are managed by pyelolithotomy are those having a single large stone in an extrarenal pelvis, which may be removed completely without transgressing the parenchyma with its attendant risks. Others may be managed by extended pyelolithotomy or the use of pyeloscopy using a flexible nephroscope through one of the abdominal ports and employing graspers or laser dusting. For some patients, dissection of the renal pelvis can be extremely challenging as the pelvis may be inflamed with adherent fat, making demarcation of planes difficult and bloody.

In cases of calyceal diverticulum and peripherally located calyceal stones where access to the collecting system through the renal pelvis is difficult, nephrolithotomy may be performed directly in the thinnest part of the parenchyma with minimal bleeding and without any vascular clamping. An intraoperative ultrasound probe may be used to locate the stone and plan the incision (113). At the moment, the use of robotic surgery is determined by availability of the robotic armamentarium, individual skills, logistics, and, especially, the cost constraints. There is a lack of available data especially in pediatrics as the majority of the experience to date is in adults and retrospective. However, it is quite possible that future generations will be more comfortable and have more access to robotic surgery than is generally the case in most pediatric urology units today.

## Vesical Stones

Most vesical stones can be managed endoscopically via the urethra [perurethral cystolithotripsy (PUCL)] and percutaneous cystolithotripsy [percutaneous (suprapubic) cystolithotripsy (PCCL)] (114).

### Perurethral Cystolithotripsy

The miniaturization of cystoscopes and URSs has made it possible to manage bladder stones up to 2 cm transurethrally with 100% clearance and minimum complications. Pneumatic (mechanical) lithoclast is a cost-effective technology and can safely be used through a 7 or 8 FG URS (115). Lasers are costly and take longer to disintegrate the same-sized stone; however, they can be used through the fine caliber miniscope and can thus be very safely used in babies <1 year of age. Postoperatively, relief of symptoms, attainment of normal voiding, and a normal pre- and post-void ultrasound of the bladder confirm a successful outcome.

### Percutaneous (Suprapubic) Cystolithotripsy

PCCL has gained popularity as a quick and safe minimally invasive procedure, especially for large stones between 2 and 3.5 cm, and even in smaller stones, PCCL is applicable where there is limited or no urethral access and management is difficult transurethrally with risks of failure, long anesthesia and surgical time, and urethral injury. PCCL has a low morbidity and complication rate, better cosmetic outcome, shorter hospital stay, and postoperative urethral catheterization compared with open cystolithotomy (116).

**Figure 1:**
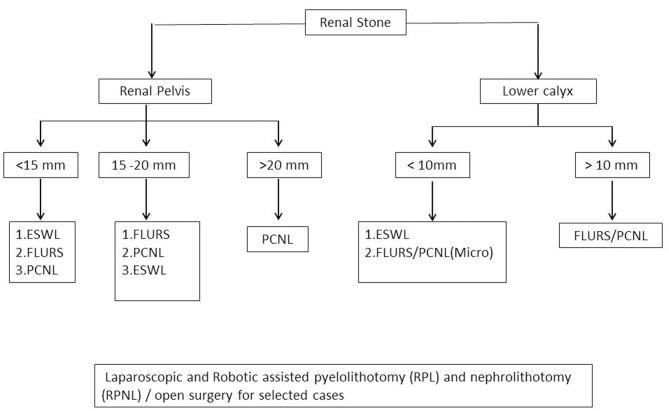
Minimally invasive surgery for renal stone management algorithm.

**Figure 2:**
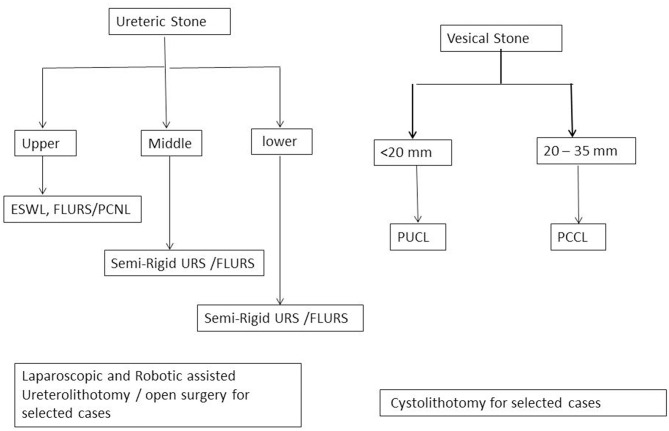
Minimally invasive surgery for ureteric and vesical stone management algorithm.

Note: Management algorithm for renal, ureteric, and vesical stones are presented in Figures 1, 2.

## Author Contributions

All authors contributed to the conception of this review. SS, SA, and BA conducted the review of the literature. SS and SA wrote the manuscript.

### Conflict of Interest Statement

The authors declare that the research was conducted in the absence of any commercial or financial relationships that could be construed as a potential conflict of interest.
